# Global travel patterns and risk of measles in Ontario and Quebec, Canada: 2007–2011

**DOI:** 10.1186/s12879-015-1039-0

**Published:** 2015-08-18

**Authors:** Sarah E. Wilson, Kamran Khan, Vladimir Gilca, Jennifer Miniota, Shelley L. Deeks, Gillian Lim, Rose Eckhardt, Shelly Bolotin, Natasha S. Crowcroft

**Affiliations:** Public Health Ontario, Toronto, ON Canada; Dalla Lana School of Public Health, University of Toronto, Toronto, ON Canada; St Michael’s Hospital, Department of Medicine, Division of Infectious Diseases, Toronto, ON Canada; Centre for Research on Inner City Health, Keenan Research Centre, Li Ka Shing Knowledge Institute, Toronto, ON Canada; Faculty of Medicine, Division of Infectious Diseases, University of Toronto, Toronto, ON Canada; Institut national de santé publique du Québec, Québec, QC Canada; Université Laval, Laval, QC Canada; Department of Laboratory Medicine and Pathobiology, University of Toronto, Toronto, ON Canada

**Keywords:** Epidemiology, Measles, Outbreaks, Surveillance, Travel, Vaccine-preventable diseases

## Abstract

**Background:**

In 2011 the largest measles outbreak in North America in a decade occurred in Quebec, Canada with over 700 cases. In contrast, measles activity in neighbouring province Ontario remained low (8 cases). Our objective was to determine the extent to which the difference could be explained by differing travel patterns.

**Methods:**

We explored the relationship between measles cases over 2007–2011, by importation classification, in Quebec and Ontario in relation to global travel patterns to each province using an ecological approach. Global measles exposure was estimated by multiplying the monthly traveler volume for each country of origin into Quebec or Ontario by the yearly measles incidence rate for the corresponding country. Visual inspection of temporal figures and calculation of Pearson correlation coefficients were performed.

**Results:**

Global measles exposure was similar in Ontario and Quebec. In Quebec, there was a nearly perfectly linear relationship between annual measles cases and its global measles exposure index over 2007–2011 (r = 0.99, p = 0.001). In contrast, there was a non-significant association in Ontario. The 2011 rise in Quebec’s index was largely driven by a dramatic increase in measles activity in France the same year.

**Conclusions:**

Global measles activity was associated with measles epidemiology in Quebec. Global measles exposure risk is higher in Ontario than Quebec. Differences in measles epidemiology between Ontario and Quebec from 2007–2011 are not explained by greater exposure in Quebec. A combination of alternative factors may be responsible, including differences in population susceptibility.

**Electronic supplementary material:**

The online version of this article (doi:10.1186/s12879-015-1039-0) contains supplementary material, which is available to authorized users.

## Background

Measles is the most communicable of vaccine-preventable diseases, requiring very high vaccine coverage for effective control. The Pan American Health Organization (PAHO) achieved its goal of endemic measles elimination by 2000 [1] and in Canada the last confirmed case of endemic measles virus was reported in 1997 [2–4]. In countries where elimination has been achieved, cases may still occur due to travel (importations) and some imported cases may result in limited local transmission (import-related cases). Measles epidemiology during elimination is therefore influenced by global measles activity and travel.

In Canada, measles vaccination policy and public health actions for measles control are provincial/territorial and regional responsibilities, respectively, and guidance on disease control and immunization is federal [5, 6]. Measles is reportable in all 13 of Canada’s provinces and territories (P/Ts) [2] and all have a 2 dose measles vaccine policy. These measures successfully kept the annual incidence of measles from 0.02 to 0.31 per 100,000 from 2001 to 2010, with annual numbers of cases ranging from 6 (2005) to 102 (2007) [7]. In 2011, however, the province of Quebec experienced the largest measles outbreak in the region of the Americas in a decade with 725 confirmed cases reported between January 8 and December 22, 2011. In contrast, neighbouring Ontario confirmed a total of 8 measles cases in 2011 [8]. Ontario and Quebec are Canada’s two most populous provinces with population sizes of 13.5 and 8.2 million, respectively.

Public health officials in Quebec attributed the 2011 measles outbreak to a combination of a chance superspreading event and sub-optimal measles immunity [9]. Our objective was to assess the contribution of travel patterns to the difference in measles epidemiology between the two provinces through a descriptive analysis and by exploring a new measure, the measles exposure index. We did this by comparing international travel and measles activity among the source countries of travelers arriving via commercial flights to Ontario and Quebec, to the measles epidemiology of each respective province over the period of 2007 to 2011. With the exception of the United States (US) (where measles has also been eliminated), international travel to and from Canada occurs almost exclusively by air rather than other modes of transport [10].

## Methods

Provincial reportable disease surveillance systems, the integrated Public Health Information System (iPHIS) in Ontario and the Maladies à Déclaration Obligatoire (MADO) in Quebec, provided measles cases reported between 2007 and 2011. We analysed cases meeting the provincial confirmed surveillance case definition, comprising laboratory-confirmed measles cases plus cases with clinically compatible signs and symptoms who are epidemiologically linked to a laboratory-confirmed case in both provinces [11, 12]. In Canada, laboratory confirmation requires isolation of measles virus, detection of measles virus ribonucleic acid (RNA), or a significant rise in measles immunoglobulin G (IgG) titre between acute and convalescent sera [5, 11, 12]. A positive serologic test for measles immunoglobulin M (IgM) antibody meets the criteria of a confirmed case only if there is an epidemiologic link to a lab-confirmed case, or a travel history to an area of known measles activity [5, 11, 12].

In both provinces, cases are imported if they have travelled outside Canada in the 7–21 days prior to symptom onset [5, 11, 12]. Import-related cases have acquired infection in Ontario or Quebec and are epidemiologically-linked to another case that travelled in the 7–21 days prior to symptom onset. Unknown importation status is assigned if exposure fields, case notes, comments, or client travel file make no reference to travel, and no clear epidemiological links made to other cases. Population data for crude incidence rates came from Statistics Canada, accessed through intelliHealth Ontario, and from the Institut de la statistique du Quebec [13].

We measured measles importation risk using International Air Transport Association (IATA) [14] anonymized flight itineraries of all travelers who embarked on commercial flights, including scheduled charters, from outside Canada with a final destination of Ontario or Quebec over the period of 2007–2011. Each flight itinerary encompasses data on the city where each traveler initiated their trip, along with all connecting flights en route to their final destination. Due to its anonymized nature, it is not possible to discern whether travelers are returning home or visitors.

We created a bivariate choropleth map for each province to estimate the spatial distribution of measles importation risk in Ontario and Quebec. These maps combine the country-specific measles incidence rates in 2011 from WHO, with the corresponding traveler volume from each country into Ontario and Quebec in 2011.

An ecological analysis quantitatively compared the cumulative risk of measles importation between Ontario and Quebec using a measles exposure index derived by multiplying the monthly traveler volume for each country into Quebec or Ontario by the annual measles incidence rate for the corresponding country, obtained from WHO. As monthly measles incidence is not available for many countries, the annual country-specific measles incidence rate was used as a proxy for each month of the relevant year. The formula for the index is shown in Box 1. For sensitivity analysis, the index was calculated for travel from France to Quebec for 2011 using monthly traveler volume and monthly measles incidence rates obtained from the European Centre for Disease Control. We assessed seasonality in Quebec and Ontario using temporal graphs illustrating monthly provincial measles activity from 2007–2011. The association between the annual number of importations and the annual global measles exposure index for each province was evaluated using Pearson correlation coefficients in STATA (version 12.1). We also did this comparing the monthly number of importations and the monthly measles exposures index value for each province, with and without a time lag of one month given that the maximum incubation period of measles is 21 days. In Quebec we calculated the Pearson correlation coefficient between monthly imported cases in 2011 and the measles exposure index related to travel from France. We explored the impact of measles cases of unknown source on the degree of association in all analyses. We made a multi-year comparison between Ontario and Quebec using monthly indices summed from 2007 to 2011 to provide an aggregate value. Finally, we identified individual countries with the greatest influence on the value of the 2011 global index of exposure for both provinces.

As we used aggregate routine surveillance data, research ethics committee approval was not required under the *Ontario Agency for Health Protection and Promotion Act* [15]. In Quebec, the Research Ethics Board of the Laval University Research Hospital Center provided an exemption letter, and all 18 Regional Directors of Public Health provided permission for the disclosure of aggregate measles data to the study team. All analyses were conducted on aggregate, rather than individual level data, routinely collected for the purposes of measles surveillance; thus, individual level informed consent was not obtained.

## Results

Between 2007 and 2011, the annual number of confirmed measles cases in Ontario ranged from a low of zero in 2007 to a peak of 58 cases in 2008 (Table 1). Between March and June 2008, an outbreak involving 54 cases occurred in southern Ontario. Although the source case was never determined, the outbreak was suspected to be import-related because of common exposure to a major tourist destination in Toronto and the identification of measles genotype D8 [16], associated with endemic transmission in the Southeast Asian and Eastern Mediterranean Regions [17]. Nevertheless, cases were classified as having an unknown source. In the province of Quebec, the annual number of confirmed cases ranged from 1 in 2008 to 725 in 2011 (Table 2). The epidemiologic characteristics of the 2011 outbreak are described in detail elsewhere [9]. Over the period of 2007–2011, 68.3 % (56/82) cases in Ontario and 3.1 % (26/832) cases in Quebec were of unknown source (Tables 1 and 2). In Ontario, 54 of the 56 cases with unknown source were from the same 2008 outbreak.

**Table 1:** Confirmed Ontario measles cases by importation status and crude measles incidence rate per 100,000 population, 2007-2011

	2007		2008		2009		2010		2011		Total
Case classification	Cases	Crude rate	Cases	Crude rate	Cases	Crude rate	Cases	Crude rate	Cases	Crude rate	Cases
Imported	0	0.00	3	0.02	1	0.01	4	0.03	4	0.03	12
Import-related	0	0.00	0	0.00	5	0.04	5	0.04	4	0.03	14
Unknown	0	0.00	55	0.43	1	0.01	0	0.00	0	0.00	56
Total	0	0.00	58	0.45	7	0.05	9	0.07	8	0.06	82

**Table 2:** Confirmed Quebec measles cases by importation status and crude measles incidence rate per 100,000 population, 2007-2011

	2007		2008		2009		2010		2011		Total
Case classification	Cases	Crude rate	Cases	Crude rate	Cases	Crude rate	Cases	Crude rate	Cases	Crude rate	Cases
Imported	0	0.00	1	0.01	2	0.03	3	0.04	17	0.21	23
Import-related	87	1.14	0	0.00	4	0.05	1	0.01	691	8.67	783
Unknown	9	0.12	0	0.00	0	0.00	0	0.00	17	0.21	26
Total	96	1.25	1	0.01	6	0.08	4	0.05	725	9.10	832

**Fig. 1:**
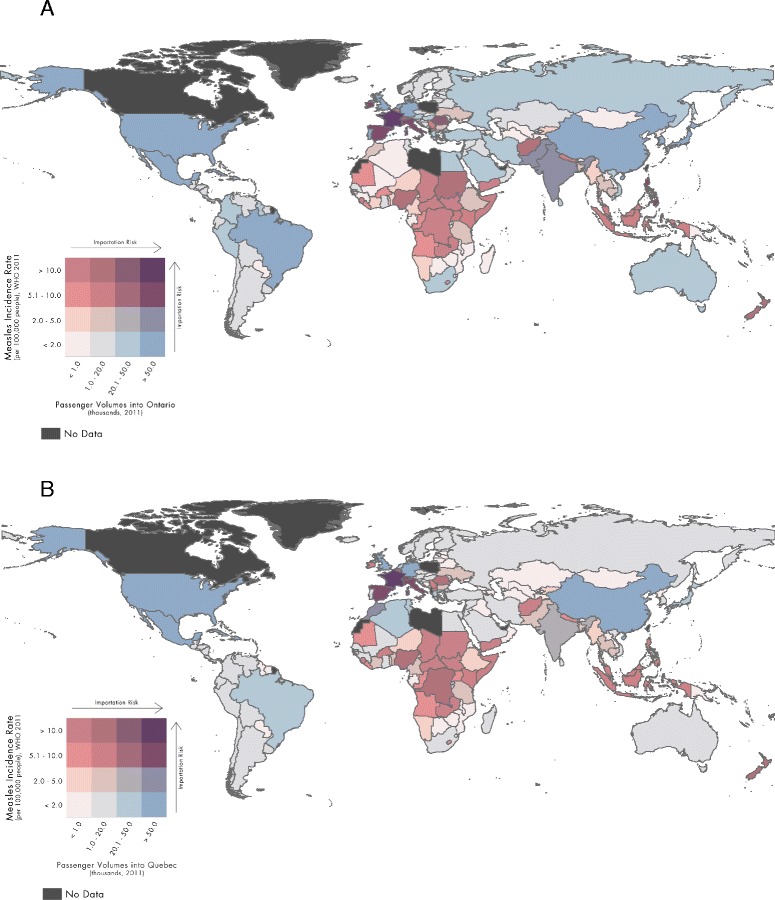
**a** Global Risk of Measles Importation into Ontario, Canada, 2011. **b** Global Risk of Measles Importation into Quebec, Canada, 2011. Data Source: WHO/World Bank, 2011; IATA, 2011

In 2011, the measles exposure index was 117 for Ontario and 151 for Quebec. Countries presenting the greatest risk of exposure to measles, on the basis of national measles incidence and corresponding passenger volume, were similar for both provinces although the index values differed (Fig. 1a and b). The top three countries contributing to Ontario’s 2011 measles exposure index (index values in parentheses) were: France (34.4), Italy (15.4) and the United Kingdom (8.5). The rank order (and values) for Quebec in 2011 were: France (107.4), Italy (9.1) and Spain (6.2). Although France was the greatest contributor to the exposure index for both provinces in 2011 it was much more influential for Quebec accounting for 71 % of the index in that province, as compared to 29 % in Ontario.

In both provinces, regular seasonal travel patterns were observed with traveler volume peaking annually in the summer months with a smaller peak in March coinciding with school holidays (Additional file 1: Figure S4A and Additional file 2: Figure S4B). The maximum monthly inbound traveler volumes are approximately twice as high in Ontario as compared to that observed in Quebec. Over the summer months of 2010 and 2011, the monthly inbound volume into Ontario was more than 800,000 passengers, with approximately 400,000 travelers per month flying into Quebec. No obvious temporal relationship existed between monthly inbound traveler volumes and measles cases in either province.

**Fig. 2:**
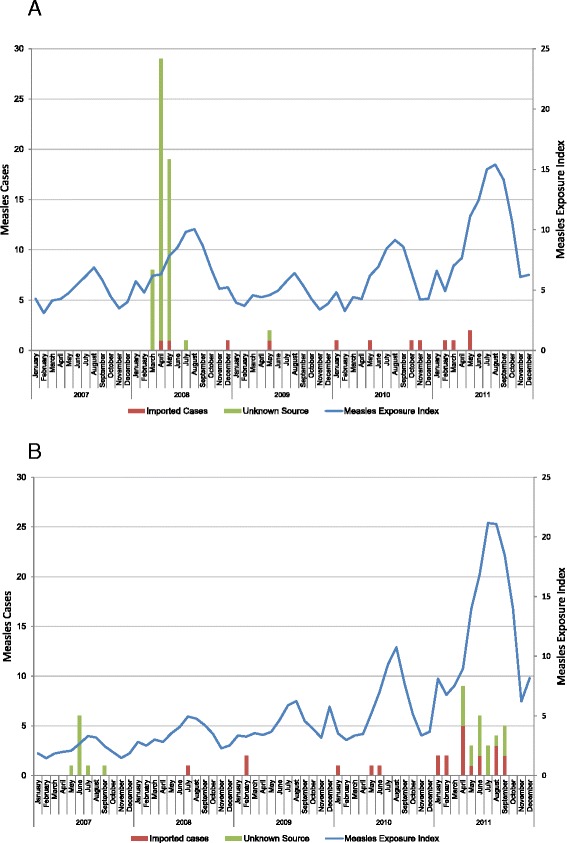
**a** Ontario measles cases (importations and unknown source) and global measles exposure index values, 2007–2011. **b** Quebec measles cases (importations and unknown source) and global measles exposure index values, 2007–2011

Comparing provincial measles activity in relation to each province’s respective global measles exposure index (Fig. 2a and b) shows fluctuation over the period of 2007–2010, with an increase in 2011. The annual values for the measles exposure index for the two provinces and the mean and range of country-specific values are shown in Table 3. The cumulative measles exposure index over the study period was 386 for the province of Ontario, and 335 for Quebec suggesting that Ontario has a slightly greater overall global exposure to measles importation than Quebec. Over the same period there were a total of 13 measles importations in Ontario and 23 importations in Quebec (Table 1). If both imported and unknown cases are combined, a total of 68 cases were reported in Ontario and 49 in Quebec over the period of 2007–2011. The annual measles exposure index correlated non-significantly with annual measles importations in Ontario (Pearson correlation coefficient, (r = 0.75, p = 0.15)) but correlated almost perfectly in Quebec (r = 0.99, p = 0.001). When comparing the degree of association between monthly measles exposure index and monthly measles importations, the strength of the relationship was attenuated but remained significant in Quebec (r = 0.48, p = 0.0001) and remained non-significant in Ontario (r = 0.04, p = 0.79). The lagged monthly analyses demonstrated similar findings for both provinces. Adding cases of unknown source to importations resulted in small changes to the coefficients in all analyses examining monthly data but did not change the significance of any findings.

**Fig. 3:**
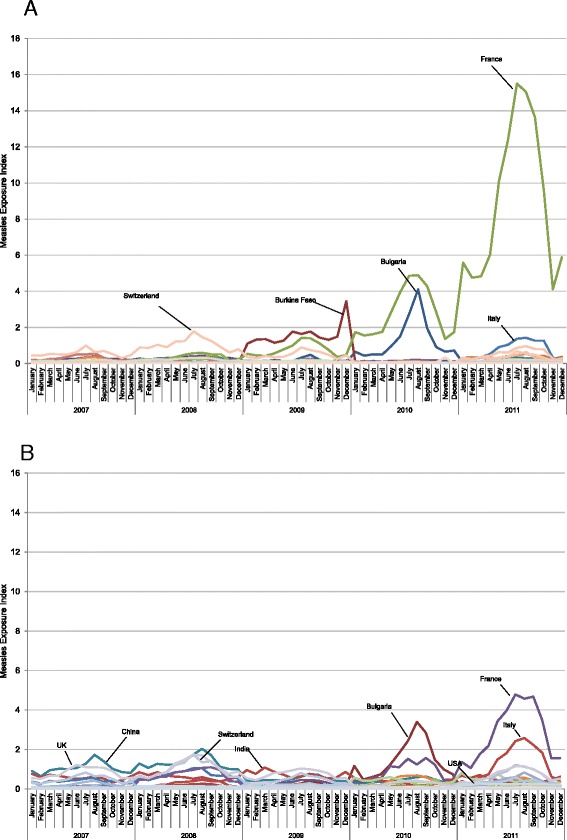
**a** Measles exposure index for Quebec by source country of arriving travelers, 2007–2011. **b** Measles exposure index for Ontario by source country of arriving travelers, 2007–2011

**Table 3:** Measles exposure index, annual totals and country-specific values, Ontario and Quebec, 2007-2011

Province	Year	Total	Country-specific values		
			Minimum	Maximum	Mean
Ontario	2007	56.9	0.0	12.7	0.32
	2008	85.1	0.0	16.1	0.47
	2009	55.2	0.0	9.1	0.31
	2010	71.0	0.0	18.0	0.39
	2011	117.4	0.0	34.4	0.65
	2007-2011	385.5	0.0	49.7	2.14
Quebec	2007	26.2	0.0	6.6	0.15
	2008	41.0	0.0	12.9	0.23
	2009	51.4	0.0	19.0	0.29
	2010	65.4	0.0	33.4	0.37
	2011	151.3	0.0	107.4	0.85
	2007-2011	335.3	0.0	154.5	1.87

In both Ontario and Quebec, the global measles exposure index increased greatly in 2011. Figure 3 depicts the most influential countries of origin in driving the value of the global measles exposure index for Quebec (Fig. 3a) and Ontario (Fig. 3b) over the study period. In both provinces, the rise in the index in 2011 was mainly driven by a measles epidemic in France, with greater influence in Quebec than in Ontario due to much larger passenger volumes. In 2011, France reported 14,966 measles cases, resulting in the highest crude incidence rate in the European Union at 23.0 cases per 100,000 population [18]. A measles exposure index calculated specifically for travel from France in 2011 correlated poorly with monthly case counts of measles importations in Quebec (r = 0.34, p = 0.28). The coefficient increased minimally when importations plus cases of unknown source were examined (r = 0.38, p = 0.23). Over the course of 2011, 4 measles importations occurred in Ontario (zero with an unknown source), compared with 17 importations to Quebec and an additional 17 measles cases of unknown source of acquisition (Tables 1 and 2).

## Discussion

Measles activity in areas from which there is frequent travel to Canada presents a significant challenge to sustaining elimination [2, 3]. Measles importations are expected, but the risk of importation is not constant over time. Over the course of 2013 and into 2014, Canada has observed increased numbers of measles importations and import-related cases, reflecting the extent of global measles activity [19–21]. Only one of these cases occurred in Quebec (one case in 2013 and zero measles incidence in 2014) [Vladimir Gilca, personal communication]. In contrast, Ontario observed a total of 15 cases in 2013 [22] and 22 in 2014 [Shelley Deeks, personal communication]. The public health actions to control measles and subsequent import-related cases are costly [23].

Our analysis suggests that the risk of measles importations can be estimated through measles surveillance in countries with close travel links, and quantified through the calculation of a measles exposure index. The findings are consistent with those of a study of measles importation into the United States via airline travelers that estimated 24 importations per year in the United States, very similar to the observed frequency of importations of 28 per year [24]. This analysis found a weaker association between the global measles exposure index and measles importations in Ontario than in Quebec, with several possible explanations. Firstly, the quality of measles surveillance data could be lower in Ontario, but it is unlikely that a large outbreak would go undetected. Other possible explanations for a lower risk of measles outbreaks in Ontario include higher immunity due to a combination of higher vaccine uptake and greater proportion of immigrants from measles endemic countries [25], who are more likely to have immunity from natural infection. Differences in measles susceptibility are unlikely to be explained by differing age structures as the proportion of the population ≥ 40 years of age in 2011 was similar (49.5 % in Ontario, 51.8 % in Quebec) [13, 26]. Individuals born prior to 1970 are likely to be immune [5].

The 2011 Quebec outbreak illustrates the importance of understanding the risk of local transmission following measles importation. Key to this is having high quality systems of coverage and population susceptibility monitoring. During the 2011 Quebec outbreak, because immunization coverage was thought to be high enough to prevent sustained transmission, public health authorities initially focused on offering vaccine or immunoglobulin to individuals at high risk of measles complications and unvaccinated close contacts, rather than initiating wide-scale supplementary immunization [9]. Provincial coverage estimates among 28-month–old children in Quebec reported 90 % 2 dose measles coverage and 97 % 1 dose coverage, with similar estimates at a regional level [27]. This is similar to coverage estimates in Ontario, where 2-dose measles vaccine coverage was 89 % among 7-year-olds and 95 % among 17-year-olds in the 2011/12 school year [28]. Lower coverage among the student population (85 % with written documentation of receipt of 2 doses, with additional susceptibility among 1 dose recipients), and a postulated superspreader event within this community may have played a crucial role in the outbreak [9]. An additional explanation to the more limited extent of import-associated cases in Ontario may be the Immunization of School Pupils Act, provincial legislation that enables un-immunized students to be excluded from school during an outbreak [29].

The strengths of this assessment include the availability of detailed measles surveillance data from Canada’s two most populous provinces, and the integration of these data with comprehensive international travel data. Limitations include that this is an ecological analysis, and our single risk measure combined travel patterns and measles incidence in the source country. We cannot discern from IATA data whether travelers are visitors or Canadians returning home from abroad, the length and nature of the travel, nor the ages of travelers. These attributes influence the likelihood of susceptibility and infection and would help refine our risk estimates if available. We did not adjust for the population size of the two provinces as our outcome of interest was the number of measles importations. Finally, cross-border non-air travel from the US into Canada was not included, but is unlikely to be important since measles is eliminated in the US.

A further limitation is that WHO provides only annual country-specific measles incidence rates, while traveler volume is available monthly. Monthly measles incidence would have allowed for a more granular measurement of global measles exposure for each province, including detecting monthly changes in risk. Finally, this assessment would have been bolstered by an accompanying analysis of measles susceptibility between the two provinces. Future directions for research involving the measles exposure index should expand on the descriptive, proof of concept work, described here and test the performance of the index in predicting the number of measles importations using statistical modelling, controlling for population measles susceptibility and other important covariates, and using an expanded dataset including a larger number of importations.

## Conclusions

The 2011 measles epidemic in France may have been an important cause of the Quebec outbreak, but our findings indicate that characteristics of the provincial measles susceptibility were more important in determining outbreak size. Over 2007–2011 Ontario had a higher degree of global exposure to measles, with relatively few cases, suggesting high herd immunity. Improvements in measles surveillance data quality, vaccine coverage assessment, and the establishment of a measles sero-epidemiology program in Canada [30], would help elucidate the risk importations pose to sustaining measles elimination in Canada. Future work to examine the performance of the measles exposure index in predicting measles cases in the post-elimination era is warranted.

## Additional files

Additional file 1: Figure S4A. Quebec measles cases (importations and unknown source) and inbound passenger volume, 2007–2011. (PPTX 18 kb) Additional file 2: Figure S4B. Ontario measles cases (importations and unknown source) and inbound passenger volume, 2007–2011. (PPTX 18 kb)

## Abbreviations

IATA: International Air Transportation Association
IgG: Immunoglobulin G
IgM: Immunoglobulin M
iPHIS: integrated Public Health Information System
MADO: Maladies à Déclaration Obligatoire
PAHO: Pan American Health Organization
P/Ts: Provinces and territories
RNA: Ribonucleic acid
WHO: World Health Organization
