# Supplementary material for: Global travel patterns and risk of measles in Ontario and Quebec, Canada: 2007–2011
Source: BMC Infect Dis. 2015 Aug 18;15:341. doi: 10.1186/s12879-015-1039-0 (PMC4539886; doi:10.1186/s12879-015-1039-0)
Supplement: Additional file 1: Figure S4A. — Quebec measles cases (importations and unknown source) and inbound passenger volume, 2007–2011. (PPTX 18 kb) [file 12879_2015_1039_MOESM1_ESM.pptx]

## Slide 1
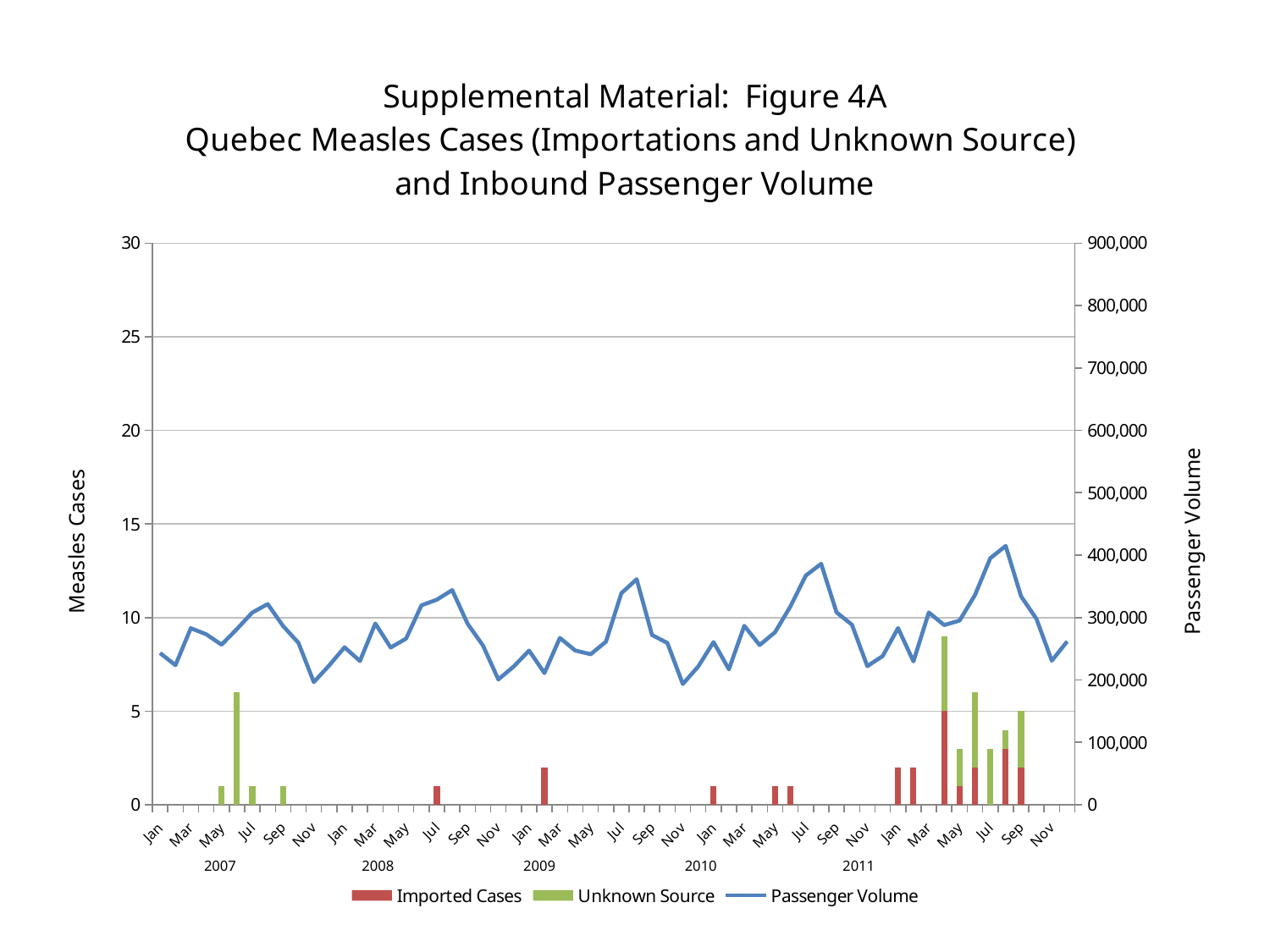

### Chart: Supplemental Material: Figure 4A
Quebec Measles Cases (Importations and Unknown Source)
and Inbound Passenger Volume
| Category | | | |
|---|---|---|---|
| Jan | 0.0 | 0.0 | 243085.0 |
| Feb | 0.0 | 0.0 | 223637.0 |
| Mar | 0.0 | 0.0 | 282884.0 |
| Apr | 0.0 | 0.0 | 273146.0 |
| May | 0.0 | 1.0 | 256398.0 |
| Jun | 0.0 | 6.0 | 281596.0 |
| Jul | 0.0 | 1.0 | 308021.0 |
| Aug | 0.0 | 0.0 | 321506.0 |
| Sep | 0.0 | 1.0 | 286432.0 |
| Oct | 0.0 | 0.0 | 259497.0 |
| Nov | 0.0 | 0.0 | 196528.0 |
| Dec | 0.0 | 0.0 | 223241.0 |
| Jan | 0.0 | 0.0 | 252252.0 |
| Feb | 0.0 | 0.0 | 230362.0 |
| Mar | 0.0 | 0.0 | 290391.0 |
| Apr | 0.0 | 0.0 | 251970.0 |
| May | 0.0 | 0.0 | 266187.0 |
| Jun | 0.0 | 0.0 | 319492.0 |
| Jul | 1.0 | 0.0 | 328590.0 |
| Aug | 0.0 | 0.0 | 343967.0 |
| Sep | 0.0 | 0.0 | 289964.0 |
| Oct | 0.0 | 0.0 | 255190.0 |
| Nov | 0.0 | 0.0 | 200788.0 |
| Dec | 0.0 | 0.0 | 221554.0 |
| Jan | 0.0 | 0.0 | 247016.0 |
| Feb | 2.0 | 0.0 | 210992.0 |
| Mar | 0.0 | 0.0 | 267306.0 |
| Apr | 0.0 | 0.0 | 247190.0 |
| May | 0.0 | 0.0 | 241114.0 |
| Jun | 0.0 | 0.0 | 261246.0 |
| Jul | 0.0 | 0.0 | 338896.0 |
| Aug | 0.0 | 0.0 | 361354.0 |
| Sep | 0.0 | 0.0 | 271988.0 |
| Oct | 0.0 | 0.0 | 259168.0 |
| Nov | 0.0 | 0.0 | 193428.0 |
| Dec | 0.0 | 0.0 | 221344.0 |
| Jan | 1.0 | 0.0 | 260640.0 |
| Feb | 0.0 | 0.0 | 217055.0 |
| Mar | 0.0 | 0.0 | 286691.0 |
| Apr | 0.0 | 0.0 | 255840.0 |
| May | 1.0 | 0.0 | 276430.0 |
| Jun | 1.0 | 0.0 | 317662.0 |
| Jul | 0.0 | 0.0 | 367153.0 |
| Aug | 0.0 | 0.0 | 386085.0 |
| Sep | 0.0 | 0.0 | 308242.0 |
| Oct | 0.0 | 0.0 | 288421.0 |
| Nov | 0.0 | 0.0 | 222108.0 |
| Dec | 0.0 | 0.0 | 238241.0 |
| Jan | 2.0 | 0.0 | 283085.0 |
| Feb | 2.0 | 0.0 | 229663.0 |
| Mar | 0.0 | 0.0 | 308076.0 |
| Apr | 5.0 | 4.0 | 287909.0 |
| May | 1.0 | 2.0 | 295141.0 |
| Jun | 2.0 | 4.0 | 335862.0 |
| Jul | 0.0 | 3.0 | 395147.0 |
| Aug | 3.0 | 1.0 | 414623.0 |
| Sep | 2.0 | 3.0 | 333992.0 |
| Oct | 0.0 | 0.0 | 297813.0 |
| Nov | 0.0 | 0.0 | 230796.0 |
| Dec | 0.0 | 0.0 | 261677.0 | 2007 2008 2009 2010 2011
