# Supplementary material for: Global travel patterns and risk of measles in Ontario and Quebec, Canada: 2007–2011
Source: BMC Infect Dis. 2015 Aug 18;15:341. doi: 10.1186/s12879-015-1039-0 (PMC4539886; doi:10.1186/s12879-015-1039-0)
Supplement: Additional file 2: Figure S4B. — Ontario measles cases (importations and unknown source) and inbound passenger volume, 2007–2011. (PPTX 18 kb) [file 12879_2015_1039_MOESM2_ESM.pptx]

## Slide 1
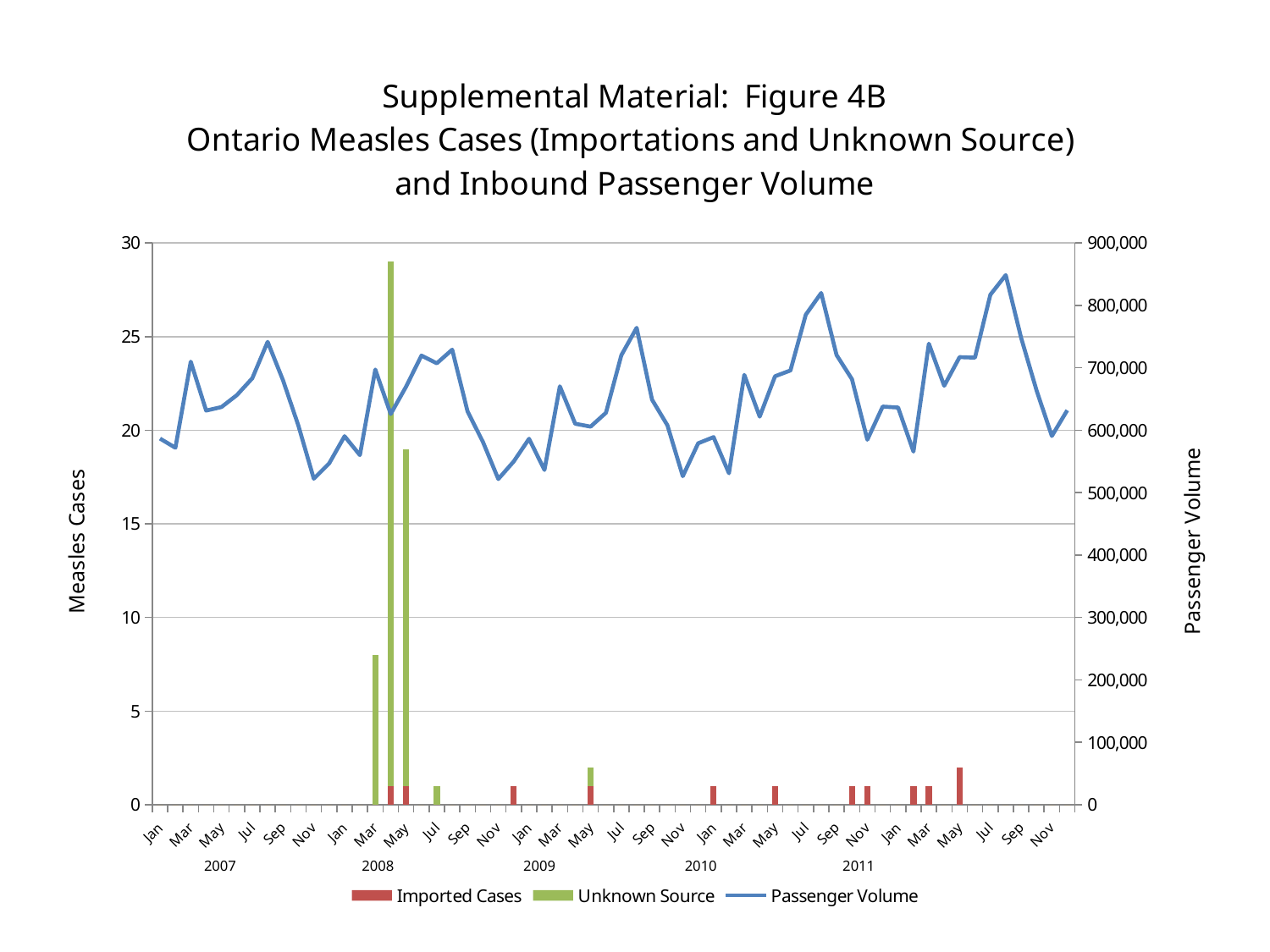

### Chart: Supplemental Material: Figure 4B
Ontario Measles Cases (Importations and Unknown Source)
and Inbound Passenger Volume
| Category | | | |
|---|---|---|---|
| Jan | 0.0 | 0.0 | 586667.0 |
| Feb | 0.0 | 0.0 | 571935.0 |
| Mar | 0.0 | 0.0 | 709913.0 |
| Apr | 0.0 | 0.0 | 631506.0 |
| May | 0.0 | 0.0 | 637228.0 |
| Jun | 0.0 | 0.0 | 656351.0 |
| Jul | 0.0 | 0.0 | 683357.0 |
| Aug | 0.0 | 0.0 | 741691.0 |
| Sep | 0.0 | 0.0 | 679974.0 |
| Oct | 0.0 | 0.0 | 607377.0 |
| Nov | 0.0 | 0.0 | 522246.0 |
| Dec | 0.0 | 0.0 | 546920.0 |
| Jan | 0.0 | 0.0 | 590492.0 |
| Feb | 0.0 | 0.0 | 560186.0 |
| Mar | 0.0 | 8.0 | 697307.0 |
| Apr | 1.0 | 28.0 | 626023.0 |
| May | 1.0 | 18.0 | 669826.0 |
| Jun | 0.0 | 0.0 | 719667.0 |
| Jul | 0.0 | 1.0 | 707277.0 |
| Aug | 0.0 | 0.0 | 729080.0 |
| Sep | 0.0 | 0.0 | 630366.0 |
| Oct | 0.0 | 0.0 | 581281.0 |
| Nov | 0.0 | 0.0 | 521748.0 |
| Dec | 1.0 | 0.0 | 549978.0 |
| Jan | 0.0 | 0.0 | 586395.0 |
| Feb | 0.0 | 0.0 | 536396.0 |
| Mar | 0.0 | 0.0 | 670330.0 |
| Apr | 0.0 | 0.0 | 610440.0 |
| May | 1.0 | 1.0 | 605852.0 |
| Jun | 0.0 | 0.0 | 627974.0 |
| Jul | 0.0 | 0.0 | 720237.0 |
| Aug | 0.0 | 0.0 | 764077.0 |
| Sep | 0.0 | 0.0 | 648943.0 |
| Oct | 0.0 | 0.0 | 608052.0 |
| Nov | 0.0 | 0.0 | 526355.0 |
| Dec | 0.0 | 0.0 | 579162.0 |
| Jan | 1.0 | 0.0 | 588991.0 |
| Feb | 0.0 | 0.0 | 531158.0 |
| Mar | 0.0 | 0.0 | 688806.0 |
| Apr | 0.0 | 0.0 | 621870.0 |
| May | 1.0 | 0.0 | 686496.0 |
| Jun | 0.0 | 0.0 | 695833.0 |
| Jul | 0.0 | 0.0 | 785146.0 |
| Aug | 0.0 | 0.0 | 819891.0 |
| Sep | 0.0 | 0.0 | 720533.0 |
| Oct | 1.0 | 0.0 | 681924.0 |
| Nov | 1.0 | 0.0 | 584840.0 |
| Dec | 0.0 | 0.0 | 637906.0 |
| Jan | 0.0 | 0.0 | 636402.0 |
| Feb | 1.0 | 0.0 | 565856.0 |
| Mar | 1.0 | 0.0 | 738715.0 |
| Apr | 0.0 | 0.0 | 671225.0 |
| May | 2.0 | 0.0 | 717182.0 |
| Jun | 0.0 | 0.0 | 716305.0 |
| Jul | 0.0 | 0.0 | 817078.0 |
| Aug | 0.0 | 0.0 | 848592.0 |
| Sep | 0.0 | 0.0 | 748225.0 |
| Oct | 0.0 | 0.0 | 664161.0 |
| Nov | 0.0 | 0.0 | 590767.0 |
| Dec | 0.0 | 0.0 | 631979.0 | 2007 2008 2009 2010 2011
